# Physarum machines imitating a Roman road network: the 3D approach

**DOI:** 10.1038/s41598-017-06961-y

**Published:** 2017-08-01

**Authors:** Vasilis Evangelidis, Jeff Jones, Nikolaos Dourvas, Michail-Antisthenis Tsompanas, Georgios Ch. Sirakoulis, Andrew Adamatzky

**Affiliations:** 1 Research and Innovation Centre “Athena”, Institute for Language and Speech Processing, Xanthi, GR67100 Greece; 2 University of the West of England, Bristol, BS16 1QY United Kingdom; 3 Laboratory of Electronics, Department of Electrical and Computer Engineering, Democritus University of Thrace, Xanthi, GR67100 Greece; 4 Unconventional Computing Ltd, Bristol, BS39 5RX United Kingdom

## Abstract

*Physarum Polycephalum* is a single cell visible by unaided eye. This is a plasmodial, vegetative stage of acellular slime mould. This single cell has myriad of nuclei which contribute to a network of bio-chemical oscillators responsible for the slime mould’s distributed sensing, concurrent information processing and decision making, and parallel actuation. When presented with a spatial configuration of sources of nutrients, the slime mould spans the sources with networks of its protoplasmic tube. These networks belong to a family of planar proximity graphs. The protoplasmic networks also show a degree of similarity to vehicular transport networks. Previously, we have shown that the foraging behaviour of the slime mould can be applied in archaeological research to complement and enhance conventional geographic information system tools. The results produced suffered from limitation of a flat substrate: transport routes imitated by the slime mould did not reflect patterns of elevations. To overcome the limitation of the ‘flat world’ we constructed a three-dimensional model of Balkans. In laboratory experiments and computer modelling we uncovered patterns of the foraging behaviour that might shed a light onto development of Roman roads in the Balkans during the imperial period (1st century BC – 4th century AD).

## Introduction

Since the emergence of new approaches in Geography (e.g. Human-Historical Geography) and Archaeology (Landscape and Settlement Archaeology) it became clear that roads or other man made networks are not simply points and lines on a map^1^. Roads-as stated by R. Witcher (1998)^2^ - are not built through an empty abstract and neutral space but, similarly to other human built features and networks, were greatly influenced by environmental and cultural variables^3^. However, analyzing the close relationship between physical landscape and human activity (or in this case to correlate geography with archaeological remains) was not always an easy task for the archaeologists. Practical limitations of macro-scale analysis^4^ restricted the ability of the archaeologists to approach the different ways that the ancient societies interacted with their environment. The employment of GIS^5^ in archaeological studies since the early 90’s seems to have provided a solution towards this direction: representation of data in layers, integration of statistical and spatial programmes and most importantly the ability to work on 3D terrains (and thus explore issues such as visual perception) gives a big advantage over static maps^6^.

GIS tools though are not the only computational or quantitative methods that archaeology has employed since the emergence of Processual archaeology in the late 50’s. There is a long disciplinary tradition of openness into methods that offer to the researchers a way to cognitively approach and understand the development of ancient societies^7^. Following this path - tradition we tried to experiment with a new kind of alternative computational method that during the last years has promised a dynamic non biased approach to issues related to the development of networks.

In the following the *Physarum polycephalum* experiments in many multidisciplinary fields are discussed and correlated with the presented research. Then, the exact experimental results of the 3D Balkans Roman roads are analyzed, while the mathematical analysis of *Physarum* graphs with weighted-edges follows. Moreover, a model based on Cellular Automata Agents is presented to provide fair match with the introduced experimental results in 3D. Finally, conclusions are drawn.

## The Physarum polycephalum experiments and limitations

*Physarum polycephalum*, a vegetative stage of acellular slime mould has repeatedly, during the last decade, demonstrated its unexpected computing abilities^8^ especially for reproducing road networks. Plasmodium is a single cell with many nuclei, which feeds on microscopic particles^9^. When foraging for its food the Plasmodium propagates towards sources of food, surrounds them, secretes enzymes and digests the food by forming a congregation of protoplasm which covers the food source. When several sources of nutrients are scattered in the Plasmodium’s range, the Plasmodium forms a network of protoplasmic tubes connecting the masses of protoplasm with the food source.

The Plasmodium’s foraging behavior can have a computation aspect^10–12^ data are represented by spatial configurations of attractants and repellents and results are represented by the structure of protoplasmic network^8^. Hence Plasmodium can solve computational problems with natural parallelism^8, 13^ e.g., related to shortest path^11^ and hierarchies of planar proximity graphs^14^, computation of plane tessellations^15, 16^, execution of logical computing schemes^17, 18^, planar shapes and concave hulls^18^, and natural implementation of spatial logic and process algebra^19^. The slime mould’s behaviour inspired a range of software implementations of novel approaches towards the design of communication and transport networks^20, 21^. The Plasmodium of *P. polycephalum* can effectively solve several “geographical” problems, such as the approximation and evaluation of human-made transport networks in several regions or countries: Africa, Australia, Belgium, Brazil, Canada, China, Germany, Iberia, Italy, Malaysia, Mexico, The Netherlands, U.K., and USA^22–26^. Each region was represented with an agar plate where oat flakes marked the major urban sites. The Plasmodium of *P. polycephalum* was inoculated in a central site (usually the capital) and the structures of protoplasmic networks were subsequently analysed. What became obvious for all the regions studied in the experiments^26^ was that the networks of the Plasmodium’s protoplasmic tubes match — at least partly — the man-made transport networks. The shape of the country (represented by the agar plate) and the spatial distribution of the urban areas (represented by sources of nutrients) can naturally play a key role in determining the exact structure of the Plasmodium network.

It was exactly this ability of the slime mould that tempted us to apply the technique so as to approximate the development of an ancient network^27^. As case study was chosen the network of roads that the Romans developed in the Balkans during the imperial period (1st century BC – 4th century AD), when the political conditions (existence of a single ideologically laden authority based on military power) favored the unification of previously culturally diverse regions. The Balkans was an important area for the imperial structure since it was not only a transition zone towards the East but, also, a border against the always threatening nomadic or semi nomadic tribes of the steppes. The establishment of Roman authority in the area (gradually from the mid of the 2nd cent BC to the mid 1st cent AD) was accompanied by the creation of an extended road network that secured the fast movement of troops towards the Danubian border but also commerce. A great part of this network naturally preexisted in the form of smaller local network of roads. Yet the unification of these small networks and their connection to the broader road network in Italy and Asia Minor was clearly a Roman accomplishment. The basic axis of this network were two great roads that originally developed as military avenues. The first (Via Egnatia) crossed the south Balkans (coastal Macedonia and Thrace) while the other (Via Diagonalis) crossed diagonically the north and central Balkans towards Byzantion. Smaller roads along the river valleys facilitated the connection between these two great roads and secured the communication between the Danubian and the coastal areas. Excavations in different Balkan sites (Serbia, Bulgaria, Greece, Albania) have brought into light not only smaller or larger sections of these ancient roads but also remains of an extended infrastructure consisted by changing stations, forts, hostels and bridges^27^.

This is a field which is thoroughly documented with new evidence (occasionally with the use of GIS or aerial photography/remote sensing techniques) being constantly added to the existing bibliography by historians and archaeologists. The sustainability of such an extended network of roads for a long period of time (2nd century BC–3rd century AD)^28, 29^, came to be one of the best manifestations of the prosperity that pax romana brought to every corner of the empire^30^, allowing the free movement of persons, goods and ideas. The Roman road network was chosen as the subject of our experiment as it is a field which is thoroughly documented with new evidence (occasionally with the use of GIS or aerial photography/remote sensing techniques) being constantly added to the existing bibliography by historians and archaeologists.

As shown and analyzed in detail in our previous article^27^ the results of the laboratory experiments indicated that the development of Plasmodium in a time frame of 96 h imitated to a great extent the network of Roman roads in the Balkans. Yet despite the impressive results in the recreation of networks the experiments with *P. Polycephalum* hide a serious limitation: they are mostly executed on a flat two dimensional substratum which lacks all these geographical features that to a great or lesser extent define the development of networks^31, 32^. Especially for the Balkan region these were features that influenced not only the development of the networks but also the development of different cultural contexts.

The Balkan region^33^ is a large triangular peninsula (surrounded by the Black Sea the Aegean sea and the Adriatic sea) which narrows to a tip as it extends to the south. The most prominent physical features are the mountain ranges which cross the peninsula from roughly the NW to SE. The Balkan mountains (from where the whole peninsula is named after) running across Bulgaria from the Black Sea coast to the border with Serbia, the Rhodope mountains between south Bulgaria and Greece, the Dinaric Alps along the Dalmatian coast and North Albania and the Pindus range along the west and central Greece are the main mountain ranges which divide the peninsula into different ecological and cultural zones.

With the exception of Danube^34^, the great river that crosses the Hungarian plain towards the Romanian Black sea coast most of the rivers (Axios-Vardar, Strymon – Struma, Skhumbin, Maritsa - Evros) are relatively short and too small for navigation. However, the valleys of these rivers were important corridors of communication, invasion and trade between the coastal Aegean zone and the interior of the Balkans. In a region where mountains are omnipresent, mountain passages like the Dragoman pass (between Serbia and Bulgaria) or the Roupel pass (between Bulgaria and Greece) were and still are important communication routes. Arable land is mostly restricted along the river valleys or small coastal or inland plains with the exception of the large central Bulgaria plain south of the river Danube.

Such a terrain favored the development of cultures that were relatively isolated one from the other. Mountain passages and river valleys were the natural routes of communication, trade or invasion. However, before the incorporation of these areas into the Roman Empire the sustainability of a unified network was very difficult. Archaeological and textual evidence indicates that the Macedonian kingdom from the time of Philipp II and his successor till the late Hellenistic period sustained a rather long and organized network of roads in the southern part of the Balkans. However, it was the establishment of Roman control that for the first time brought the unification of many of the preexisting smaller local networks of roads. Towards this direction helped definitely the ability (mastered by the Roman engineering) to overcome physical barriers like the crossing of rivers but also the ability to sustain the infrastructure of such a network in a politically controlled and stabilized area. The development of the network brought a series of changes in the settlement pattern (especially in the interior of the Balkans) with new cities and towns appearing along the routes of the roads^35^. Natural geography as such clearly played a very determinant role in the decision making process of the ancient planners or builders. If then we want to consider the method as an archaeological tool it is necessary to incorporate this physical geography aspect.

## Results and Limitations

**Figure 1:**
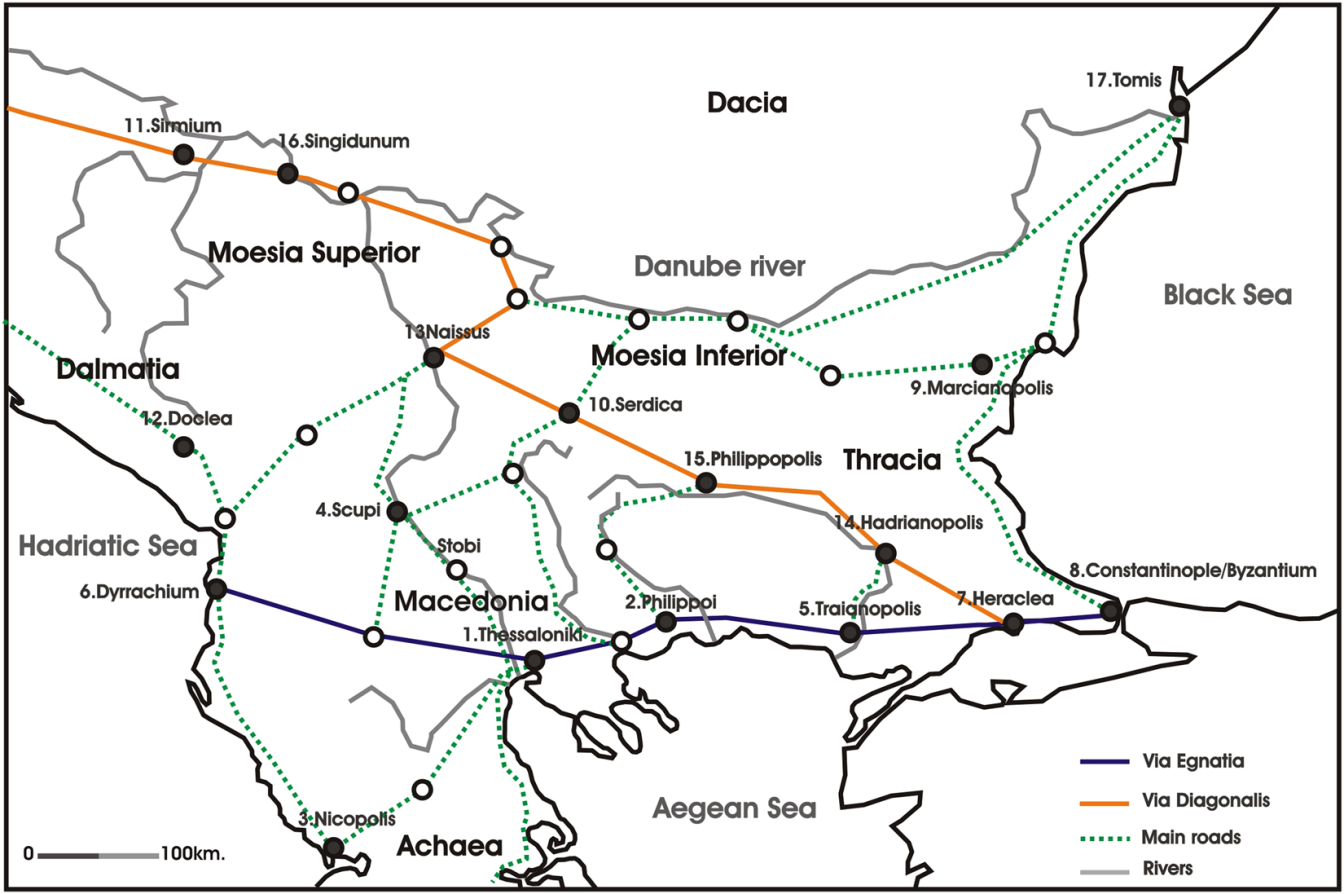
Roads and major sites in the Balkans during the imperial period (Figure prepared with Corel Draw R10 10-410 edition. Copyright 2015 V. Evangelidis. All rights reserved).

**Figure 2:**
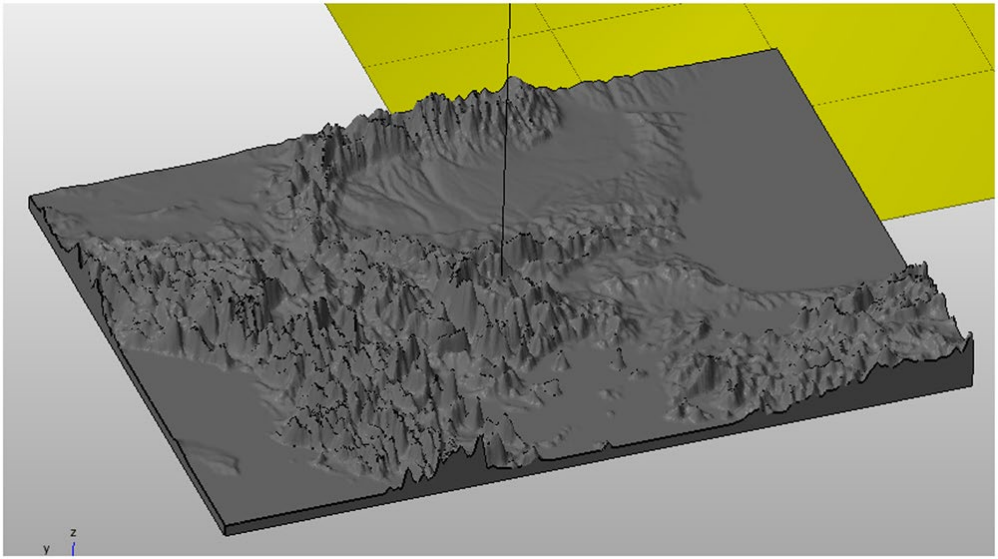
3D Balkans territory template (Terrain data is from Jarvis A., H.I. Reuter, A. Nelson, E. Guevara, 2008, Hole-filled seamless SRTM data V4, International Centre for Tropical Agriculture (CIAT), available from http://srtm.csi.cgiar.org introduced by author A.A. See text for further details).

In order to incorporate the physical geography aspect, the experimentation on a 3D terrain that assimilates the physical geography seems to be a necessity. The Plasmodium of *P. polycephalum* was cultivated in a plastic container, on paper kitchen towels moistened with still water, and fed with oat flakes. The agar plates were developed in 3D following the geomorphology of the region of the Balkans (Fig. 1), as it is depicted in the map of Fig. 2. More specifically, the proposed template was developed by PrintableGeorgraphy (http://www.printablegeography.com) and resulted to 3mm substrate with 17mm high for the tallest mountains (Fig. 2). In particular, 3D terrain of Balkans was produced as follows: The elevation data are downloaded from DIVAGIS, original source is CGIAR (http://srtm.csi.cgiar.org/). The data are projected with Mercator. The terrain was printed using Selective Laser Sintered PA 2200 with Nylon 12. Furthermore, based on their administrative and strategic position, we have selected 17 of the major urban centres of the imperial period (Middle and Late Roman Period) in the area of the Balkans (Fig. 1) which are: (1) Thessaloniki, (2) Philippoi, (3) Nicopolis, (4) Scupi (Skopje) and/or Stobi, (5) Traianoupolis, (6) Dyrrhachium, (7) Heraclea, (8) Constantinople/Byzantium, (9) Marcianopolis, (10) Serdica, (11) Sirmium, (12) Doclea, (13) Remesiana–Naissus, (14) Hadrianopolis, (15) Phillipopolis, (16) Singidunum and (17) Tomis.

**Figure 3:**
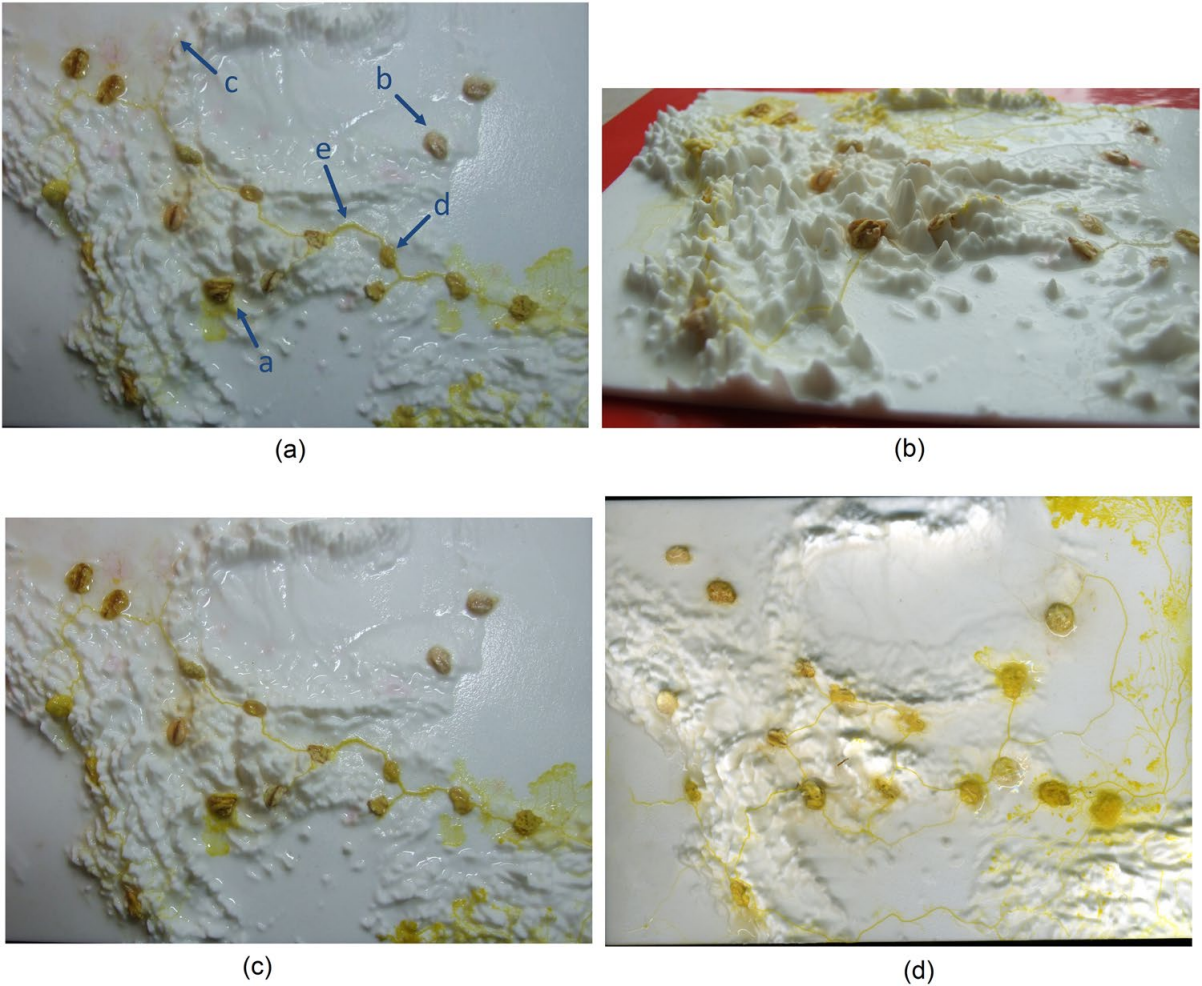
Experimental laboratory results. (**a**) A typical image of *Physarum polycephalum* growing on a non-nutrient substrate and connecting oat flakes, which represent major settlement areas U by a network of protoplasmic tubes, where a: site of inoculation, (**b**) oat flakes, (**c**) active zone, propagating in a search for nutrients, (**d**) oat flake occupied by Plasmodium, (**e**) protoplasmic tube. (Balkans territory template (Terrai): *Physarum polycephalum* growing in the 3D Balkans template and depictured in angular view. (**c** and **d**) Experimental laboratory example of north-east and then west propagation of the slime mould. Petri dishes with slime mould were scanned every 24 h.

At the beginning of each experiment an oat flake which was colonized by Plasmodium was placed in the area of Thessaloniki, which we have selected as our central site due to its strategic location between the Aegean zone and the hinterland of the Balkans. We undertook 13 experiments. The terrains were kept in closed, yet naturally ventilated, containers. The terrain was not artificially wetted or covered with any water retaining substrate but stayed in containers filled with water (terrain was positioned above the water, without water even touching edges of the terrains). Humidity is proved to be sufficient for the slime mold to propagate on a bare nylon surface [see Fig. 3]. The terrain was kept in darkness, at temperature 22–25 °C, except for observation and image recording. Configurations of Plasmodium networks were photographed with FujiFilm FinePix 6000 camera and scanned with an Epson Perfection 4490 scanner. Experimental setups with 3D terrain are illustrated in Fig. 3(a),(b),(c) and (d). More specifically, in Fig. 3(a) a typical image is provided of slime mould *P. polycephalum* growing on a non-nutrient substrate and connecting oat flakes, which represent major settlement areas U by a network of protoplasmic tubes, where a: site of inoculation, b: oat flakes, c: active zone, propagating in a search for nutrients, d: oat flake occupied by Plasmodium, e: protoplasmic tube.

To understand influence of elevations on the slime mold’s propagation, we compared our findings with conducted experiments on a flat agar gel found in our previous works. For those experiments, we have used 220 × 220 mm polystyrene square Petri dishes and 2% agar gel (Select agar, by Sigma Aldrich) as a substrate. Agar plates, about 2–3 mm in depth, were cut in shape of Balkans as mentioned in details in ref. 27. In order to reproduce the results of the biological experiments we have also introduced a CA model^36, 37^. The basic idea^27^ was to mimic the Physarum’s behavior when it propagates on a non-nutrient substrate and connects the oat flakes that represent the aforementioned Roman period sites in the Balkans. In this way, we were able to reproduce efficiently more simulation scenarios in less time and efficiently reproduce the Physarum’s behaviour that inspired us for the application of unconventional computation methods in archaeology.

Here it should be noticed that we envisaged that plasmodium of P. polycephalum will not completely ‘ignore’ 3D topography because the slime mould is gravisensitive and positively geotropic^38–41^. The experimental data showed the existence of gravisensitivity in Physarum, without suggesting a mechanism^42^. By applying inhibitors of respiration, it was possible to show in the clinostat that the receptor site of gravisensitivity may be correlated with the mitochondria^42^. Moreover, plasmodium shows morphological geopolarity, as discovered in ref. 38: the ectoplasmic wall of a slime mould tube, lying or hanging on horizontal surface, is much thinner on side closer to earth. Moreover, Benjaminson *et al*.^41^ found that the measurements of the mature stage after life-long exposure to simulated altered gravity show that the final height of the sorocarp is ultimately determined, at least partially, by the gravity environment in which development occurs. We expected that being placed on a 3D terrain with a source of nutrients slime mould would propagate towards the source of nutrients and navigate around elevations due to positive geotropism and relatively lower humidity of the elevations.

### Results of laboratory experiments

As mentioned above, the experiments that were executed on the 3D terrain are in qualitative agreement with those of our previous experiment^27^. Few hours after inoculation in Thessaloniki Plasmodium recovers from the initial shock, starts exploring its substrate, detects gradients of chemo-attractants emitted by virgin oat flakes and propagates towards them^43–46^. It has been found that after the first 24 hours *Physarum* propagates in most of the experiments either to the north and then turn east and tries to span and spans remaining settlements with its network of protoplasmic tubes to the rest of the places or to the east then north and finally to the remaining places in the 3D pattern.

In order to present more clearly our experimental results we have constructed a Physarum graph with weighted-edges. A Physarum graph is depicted as **P** = (**C,R,p**), where **C** is a set of food spots presenting the cities of the area, **R** is a set of edges representing the roads between the cities and **p** holds a probability value for each edge. Between two cities *a* and *b* we assume that there is an edge in the Physarum graph if a protoplasmic tube connection is visible between two foods representing the cities *a* and *b*. Then, this edge takes a probability value which is proportional to the appearing frequency of this edge in our experiments. For example, if we observed a protoplasmic tube connecting the cities *a* and *b* in 3 experiments, the probability of the edge (*a*,*b*) will be p(*a*,*b*) = 3/13, where 13 is the number of our experiments. We will also be dealing with threshold Physarum graphs **P**(*θ*) = (**C**,T(**R**), p, *θ*).

**Figure 4:**
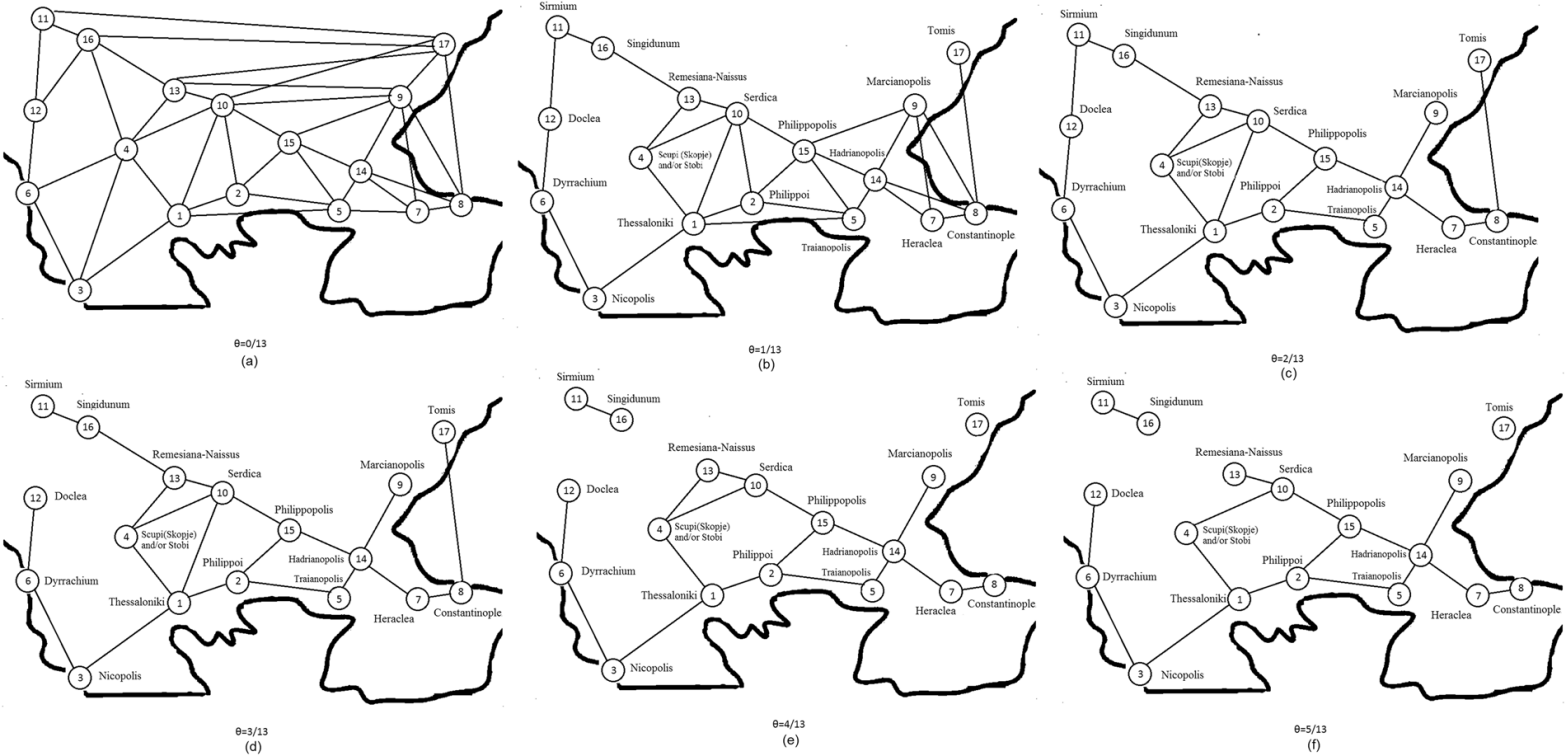
Generalized Physarum graphs **P**(*θ*) 1 ≤ *θ* ≤ 5 (Figure from Microsoft paint v. 1607 and data points from Matlab v. R2011b by author N.D.).

**Figure 5:**
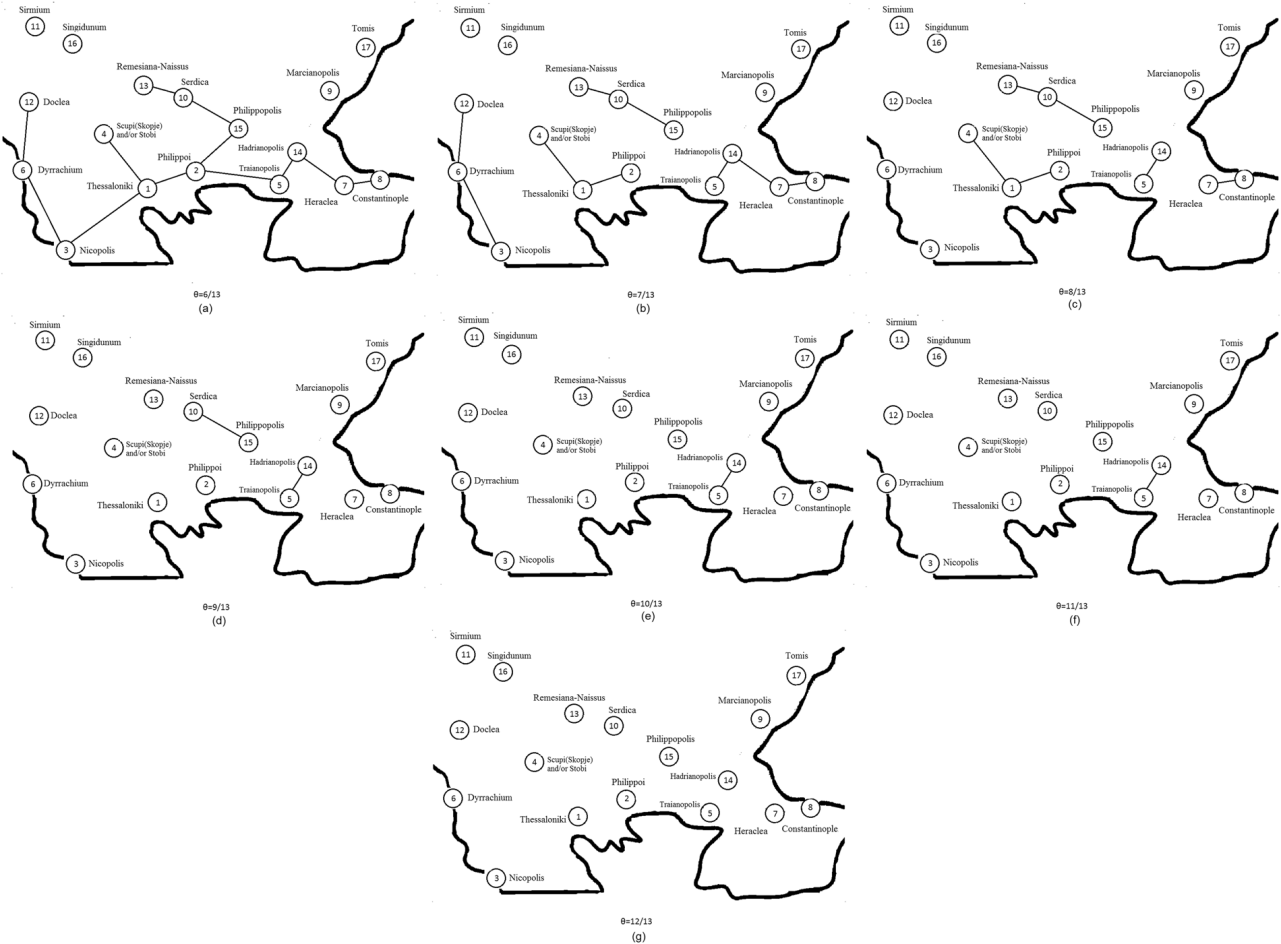
Generalized Physarum graphs **P**(*θ*) 6 ≤ *θ* ≤ 12 (Figure from Microsoft paint v. 1607 and data points from Matlab v. R2011b by author N.D.).

The threshold Physarum graph is obtained from the Physarum graph by the transformation: T(**R**) = e ∈ **R**: p(e) ≥ *θ* where in each threshold Physarum graph the respective *θ* parameter depicts the minimum frequency of the appearance of each edge. Thus, only edges observed in *θ* or more of the 13 experiments are included. So in regards to *θ*, all edges with weights less than or equal to *θ* are removed. As such, it is clear that we do not take into account the exact configuration of the protoplasmatic tubes but merely their existence. As a result, with the analysis of *θ* parameter we are able to compare the protoplasmic networks with the roman networks and proceed with their statistical analysis. From now, in the rest of our analysis, we will use the term “Physarum graph” when talking about the threshold Physarum graph. Examples of threshold Physarum graphs for various values of *θ* are shown in Figs 4 and 5, respectively.

The results are the following:

- Physarum graph becomes planar when *θ* = 2/13.
- Physarum graph becomes disconnected and node Tomis become isolated from other nodes when *θ* exceeds 3/13. Segment Sirmium-Singindunum becomes isolated from the other nodes also when *θ* exceeds 3/13.
- For *θ* = 6/13 the nodes Tomis, Marcianapolis, Sirmium, Singidunum are isolated from the rest of the Physarum graph.
- For *θ* = 7/13 Physarum graph undergoes major structure transformation. The graph splits in three isolated segments: Doclea-Dyrrachium-Nicopolis, Remesiana-Serdica-Philippopolis, Scupi-Thessaloniki-Philippoi.
- For *θ* = 10/13 all nodes are isolated except segment Traianopolis-Hadrianopolis.

**Figure 6:**
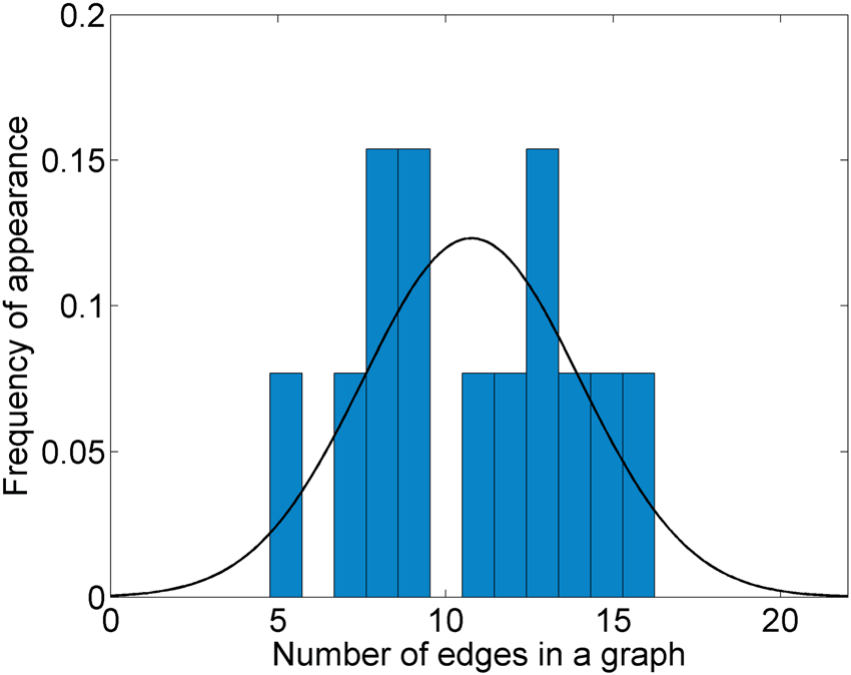
Histogram of the frequency of the amount of graph edges compared with a normal distribution with standard deviation of 3.23 and mean 10.76, respectively (Figure from Matlab v. R2016b by author M.-A.T.).

To investigate the error imposed to the biological experiments providing the Physarum graphs, the standard deviation of the number of edges in each experiment was depicted in Fig. 6. The histogram of Fig. 6. is normalized to display “relative” frequencies of number of edges in each experiment. Moreover, the normal distribution with standard deviation of value 3.23 and mean 10.76, respectively, is depicted. Note that these values are derived from the sample data set of the experiments.

## Modeling

Slime mould, as a living system, is subject to limitations of repeatability and unpredictability during its foraging, in addition to the long time it takes to form and adapt its transport networks. To aid the analysis of slime mould networks we require a computational mechanism to approximate the evolution of *Physarum* transport networks. Importantly, however, we must ensure that the networks emerge — as with slime mould — from simple, local, and ‘bottom-up’ interactions, as opposed to the ‘top-down’ network constructs formed from classical algorithmic approaches.

We use the multi-agent approach that was introduced in^47^ to approximate the emergent transport networks of slime mould. This approach has been demonstrated to be useful for approximating the biological behaviour of slime mould^48, 49^, and in developing biologically inspired unconventional computing methods^50^. A population of mobile particles is created and initialised on a 2D lattice configured to the experimental arena of the Balkans region. The diffusive medium is represented by a discrete two-dimensional floating point lattice. Particle positions are stored on a discrete lattice isomorphic to the diffusive lattice. Particles also store internal floating point representations of position and orientation which are rounded to discrete values to compute movement updates and sensory inputs. A single particle, and an aggregation of particles, are related to the *P. polycephalum* Plasmodium in the following way: the Plasmodium is conceptualised as an aggregate of identical components. Each particle represents a hypothetical unit of gel/sol interaction. Gel refers to the relatively stiff sponge-like matrix composed of actin–myosin fibres and sol refers to the protoplasmic solution which flows within the matrix. The structure of the protoplasmic network is indicated by the particle positions and the flux of sol within the network is represented by the movement of the particles. The resistance of the gel matrix to protoplasmic flux of sol is generated by particle movement collisions. For a more detailed description of the model implementation, refer to the Supplementary Information and ref. 50.

### Representation of landscape arena in the model

**Figure 7:**
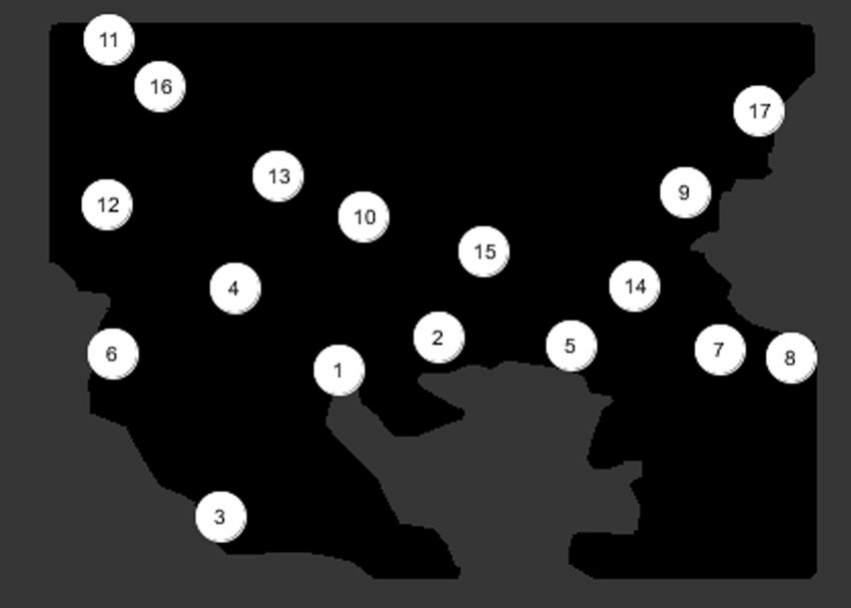
Numbers corresponding to Balkan regions (Figure and data points from custom Processing script (https://www.processing.org/) by author J.J.).

Regions are denoted by the aforementioned numerical list and correspond to the areas in Fig. 7.

**Figure 8:**
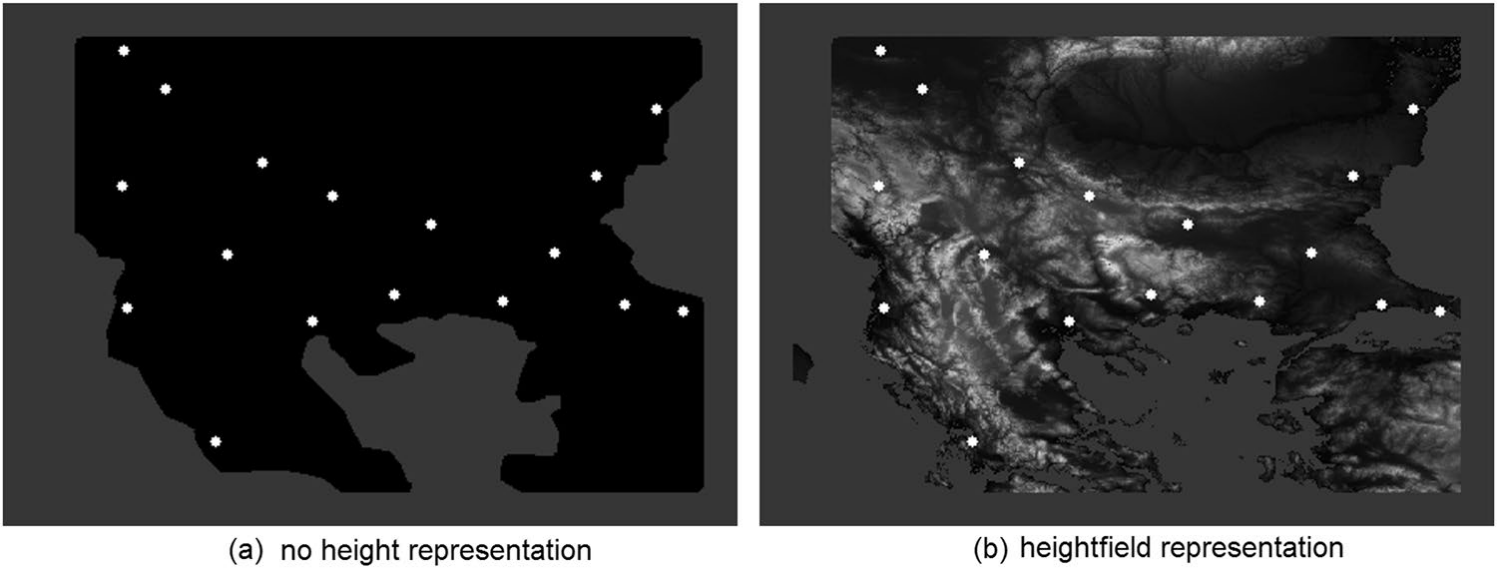
Representations of arenas used in modelling experiments. (**a**) Arena with borders of the Balkans region and no height representation, region locations represented as attractant sites (white dots), (**b**) arena represented by a greyscale topographic heightfield map where pixel intensity corresponds to landscape height, region locations represented as attractant sites (white dots) (Figure, data points and heightfield representation from custom Processing script (https://www.processing.org/) by author J.J. See text for further details).

We used two different landscape representations as the arenas for the model Plasmodium. The first has no heightfield representation of the landscape topography and contains only the borders of the region and nutrient attractant sites at the corresponding regions (Fig. 8(a)). The second representation contains the same region represented as an 8-bit greyscale heightfield map where pixel intensity corresponds to topographic height (Fig. 8(b)). This data was generated using the Whitebox Geospatial Analysis Tools program which (when specifying latitude and longitude coordinates bounding the Balkans region) builds a greyscale heightfield map based upon geological terrain sampled by satellite surveying methods (see^51^ for more information).

**Figure 9:**
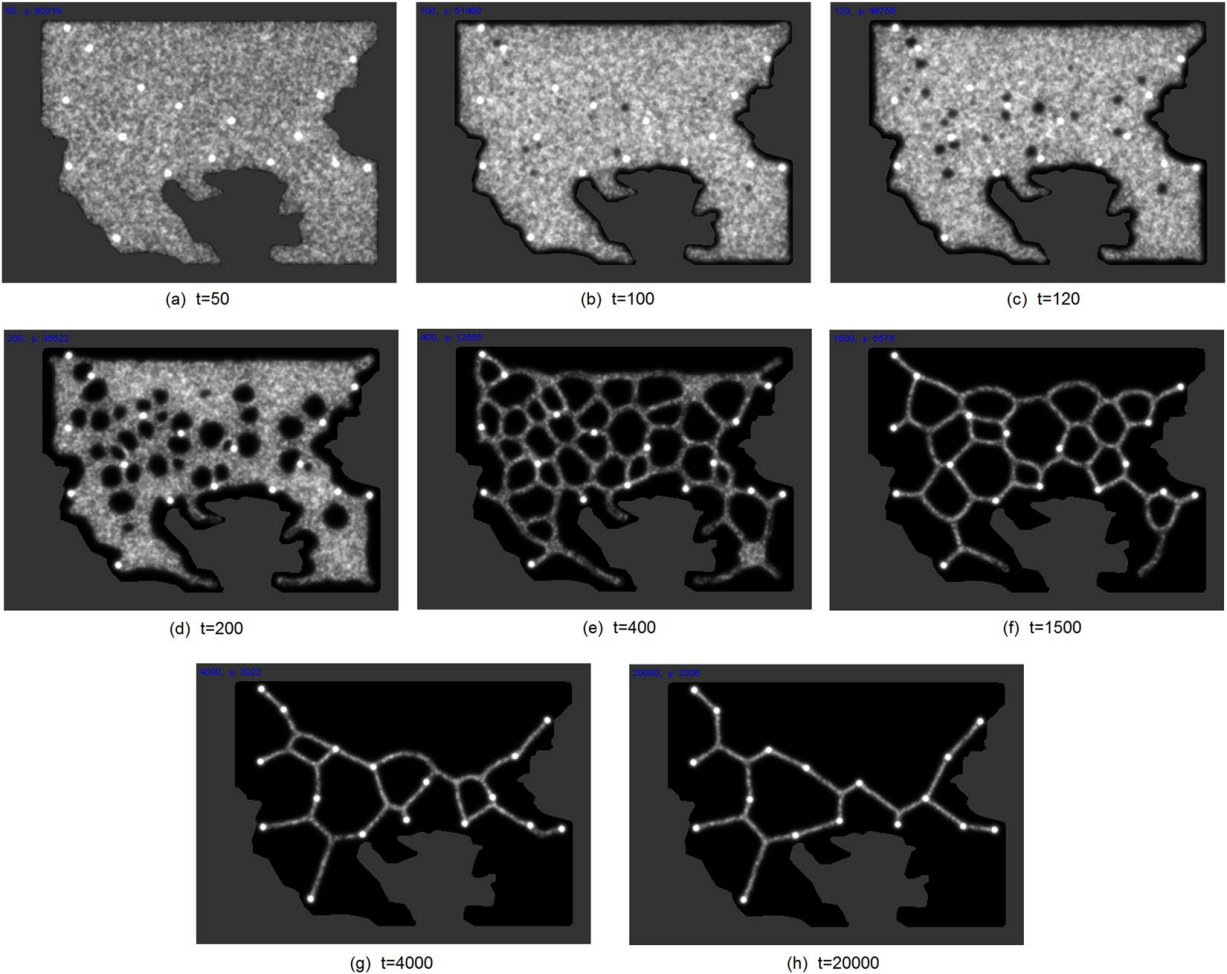
Example evolution of a single experiment with no height representation, particle trails indicated by greyscale values. (**a**) Model Plasmodium inoculated as a single mass completely occupying available locations, (**b**) as adaptation progresses, pores begin to form in the virtual material, (**c**–**e**) pores grow in size until networks are formed, (**f**–**g**) network adaptation is constrained by attraction to nutrient stimuli (bright dots), (**h**) stable network is formed (Simulation result snapshots from custom Java simulation of slime mould (https://www.processing.org/) by author J.J.).

The topology of *P. polycephalum* transport networks is, in part, influenced by unpredictable influences on its formation, for example the initial migration direction of the Plasmodium or the presence of previously laid down protoplasmic tubes. This raises the question as to whether the topology of the networks would be more regular under idealised adaptation conditions. In order to minimise this unpredictability, we initialised the simulation model with an approximation of a fully grown virtual Plasmodium. This enables us to assess what specific effect the spatial arrangement of nutrients (corresponding to Balkans region locations) had on the morphological adaptation of the virtual transport network. The results of an example evolution of the virtual Plasmodium are shown in Fig. 9. Uniform coverage was attained by populating the habitable area of the Balkans region arena with 70,000 particles.

### Network Analysis

**Figure 10:**
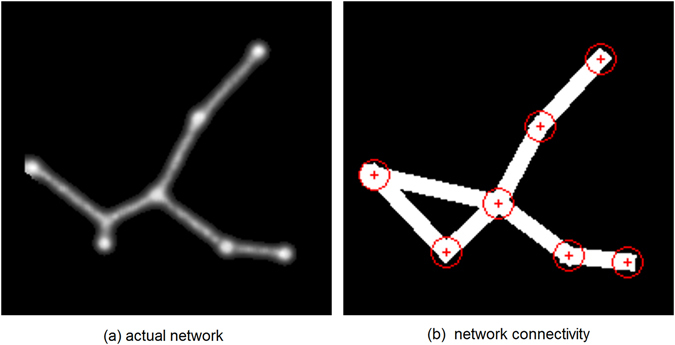
Analysis of model network method. (**a**) Fragment of model network showing trails (grey), nodes (white) and additional Steiner node, (**b**) analysed network where indirect connections via the Steiner node are interpreted as connecting the indirectly linked regions (edges as white bars, nodes circled) (Simulation result and connectivity graph from custom Java simulation of slime mould (https://www.processing.org/) by author J.J.).

The result of each experiment is a network connecting the nodes representing the regions (for example, Fig. 9(h)). Note that this network also has additional nodes (Steiner nodes) connecting the regions. A region may be directly connected to a neighbour by a single edge or it may be connected indirectly, via a Steiner node. In the analysis we consider indirectly connected regions as being directly connected if they are connected by a Steiner point (see Fig. 10 for an example).

## Modeling Results

**Figure 11:**
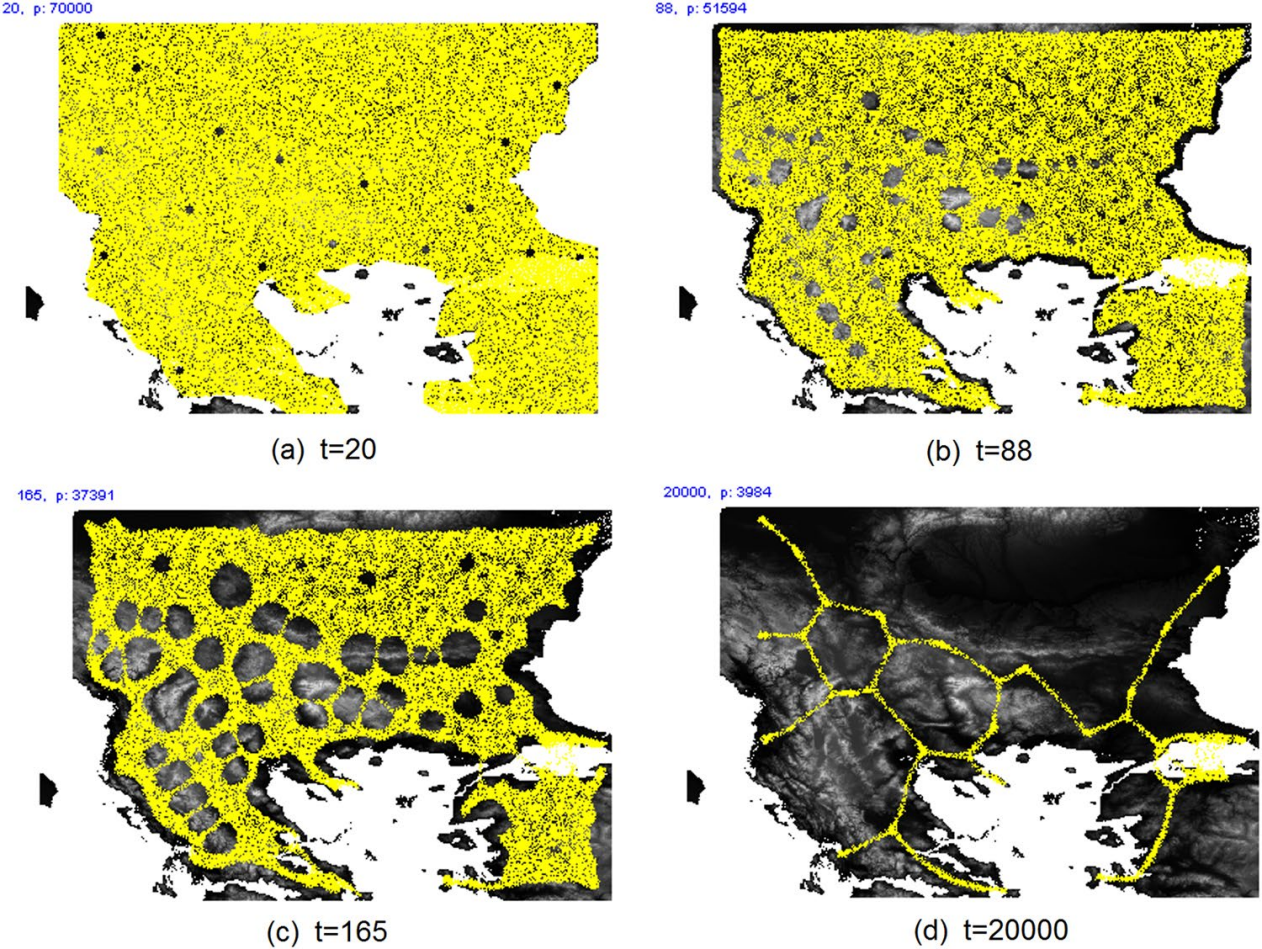
Example evolution of a single experiment under influence of landscape height, particle positions indicated by yellow (online). (**a**) Model Plasmodium inoculated as a single mass completely occupying available locations, (**b**) as adaptation progresses, pores begin to form in the virtual material, (**c**) pores predominantly form in areas of higher landscape height, (**d**) adaptation continues to minimise network (Simulation results from custom Java simulation of slime mould (https://www.processing.org/) by author J.J.).

The evolution of an experiment under the influence of landscape height is shown in Fig. 11. The evolution proceeds in a similar manner to the no-height condition but the pores in the virtual Plasmodium form predominantly at regions of greater landscape height (Fig. 11(b) and (c)). The network minimisation continues as with the no-height condition but the network adaptation is constrained by the influence of landscape height. Note that there were occasionally some spurious remnants of particle trails (for example in the bottom right corner of Fig. 11(d)). These were caused by the particle trails becoming ‘trapped’ by the terrain in enclosed regions and did not affect the network analysis as these regions were disconnected from region nodes.

**Figure 12:**
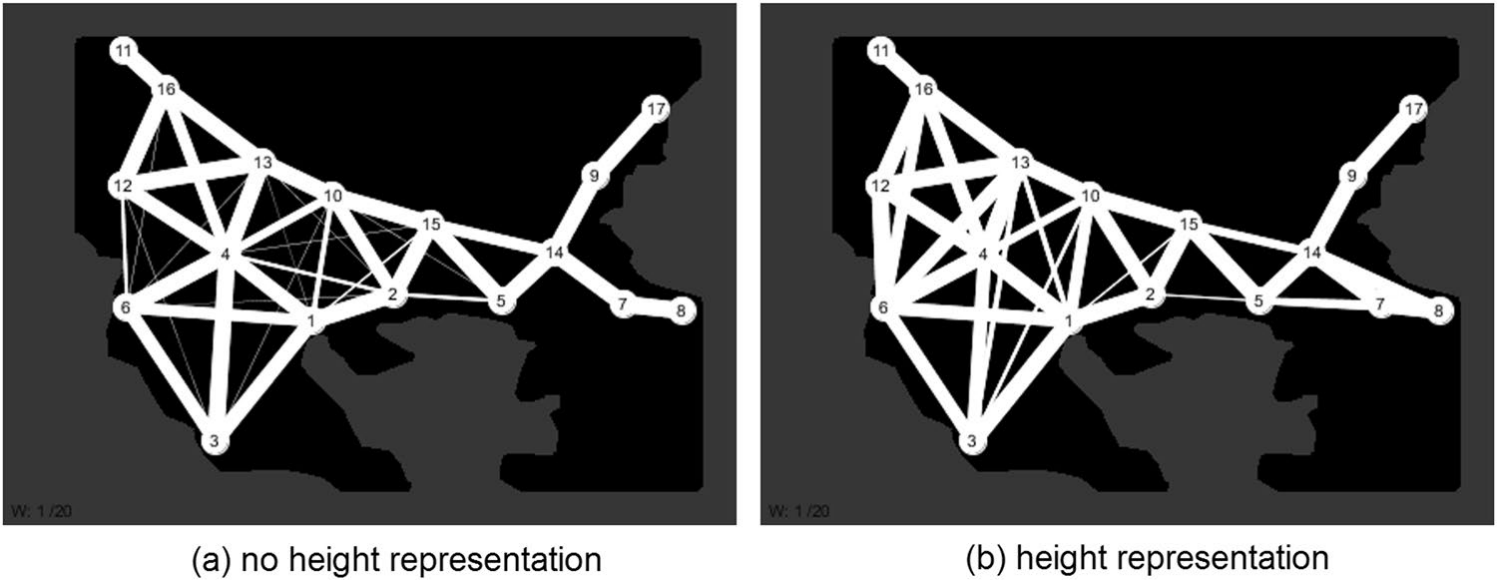
Proportional representation of edge weights denoting most frequently generated edges over 20 experiments, edge width is proportional to incidence. (**a**) No height representation condition, (**b**) heightfield representation condition (Connectivity analysis of simulation results from custom Processing script (https://www.processing.org/) by author J.J.).

Twenty experiments were performed for both no-height and landscape height conditions. Adjacency matrices were constructed from the network analysis results and the results are summarised in Fig. 12 where edge strength is proportional to edge thickness.

**Figure 13:**
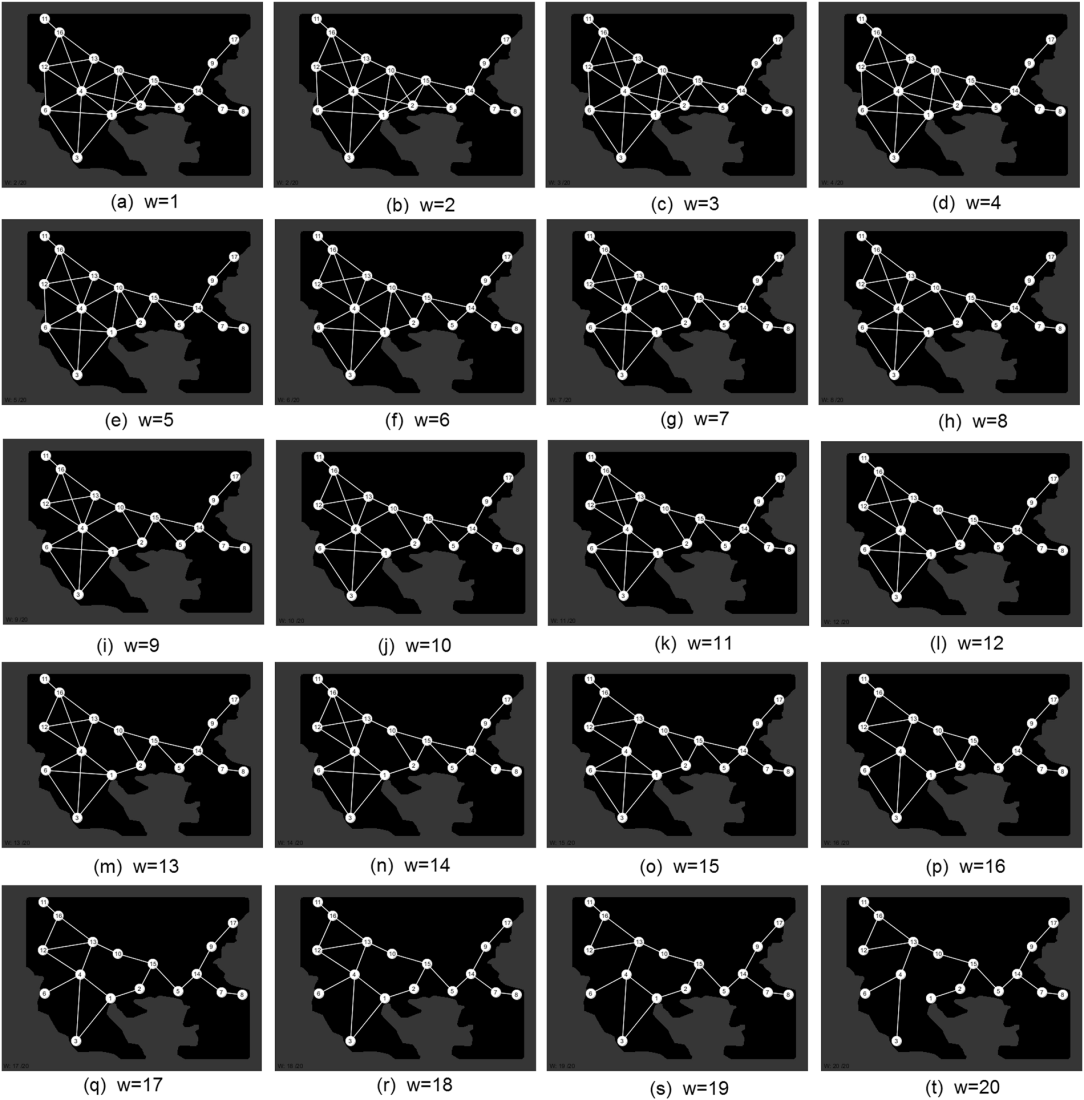
Changes in network connectivity over 20 experiments as edge weight increases, no height representation condition. (**a–t**) Graph of final connectivity corresponding to *w* value (Connectivity analysis of simulation results from custom Processing script (https://www.processing.org/) by author J.J.).

**Figure 14:**
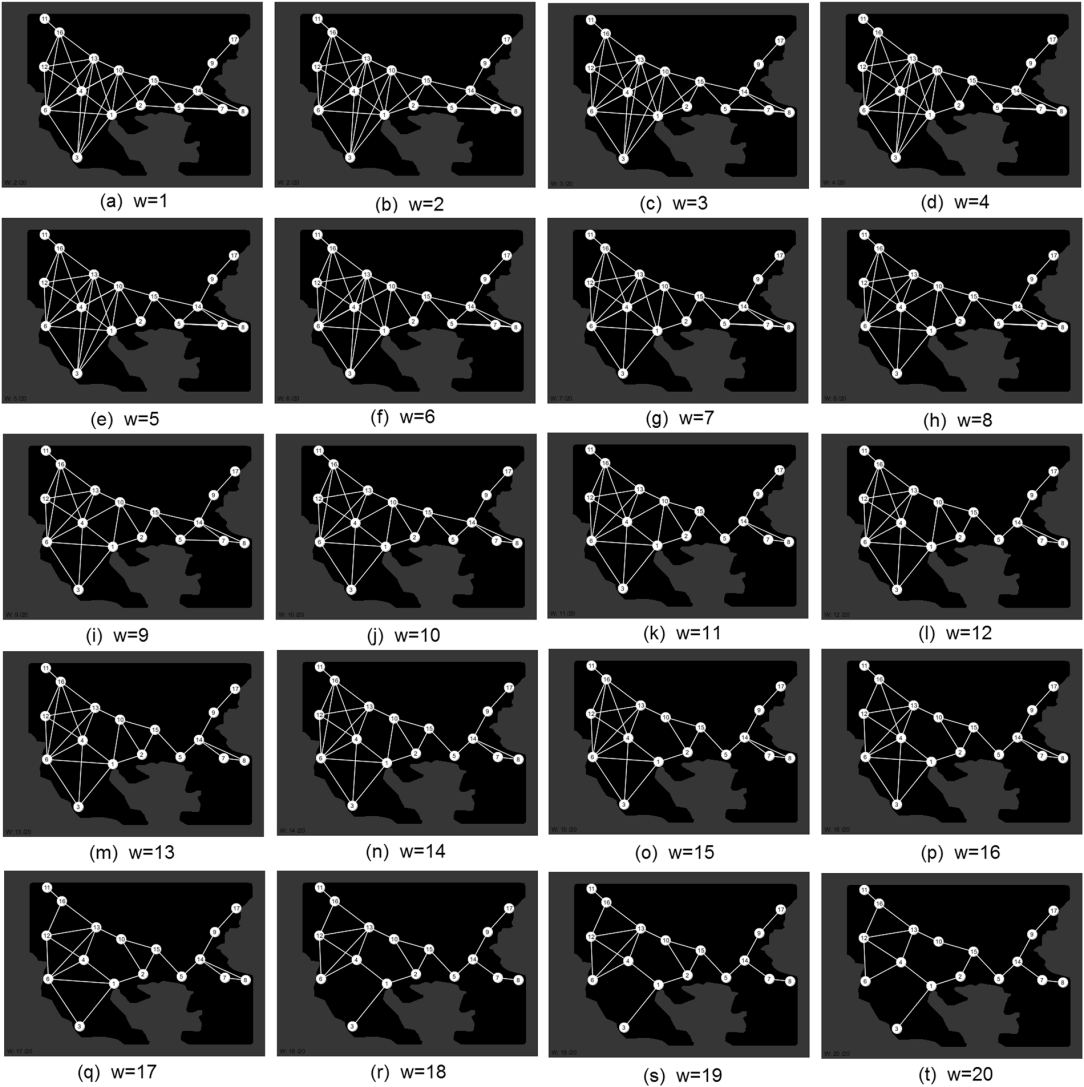
Changes in network connectivity over 20 experiments as edge weight increases, heightfield representation condition. (**a–t**) Graph of final connectivity corresponding to *w* value (Connectivity analysis of simulation results from custom Processing script (https://www.processing.org/) by author J.J.).

**Figure 15:**
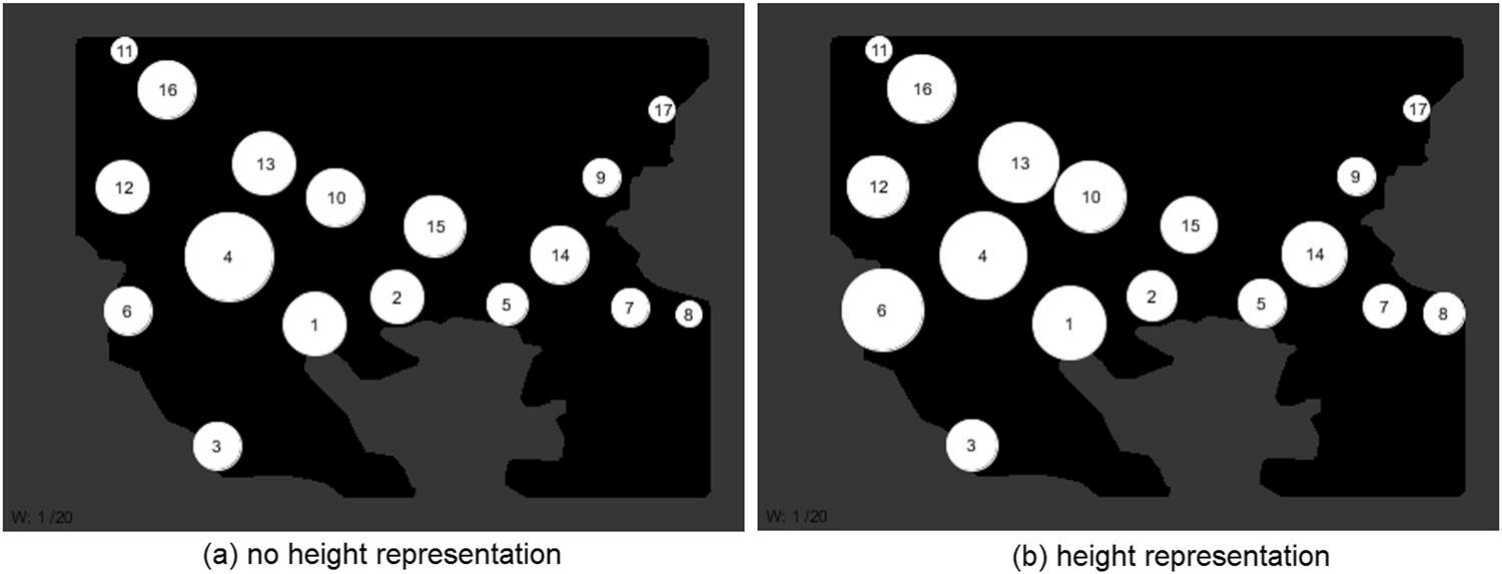
Proportional representation of region influence denoting mean degree of nodes (regions) over 20 experiments, node radius is proportional to mean degree (from Table 1). (**a**) No height representation condition, (**b**) heightfield representation condition.

The connectivity of both no-height and landscape height conditions with increasing values of the edge weight parameter *w* can be seen in Fig. 13 and Fig. 14. Numerical values of mean degree connectivity for both conditions can be seen in Table 1 which shows the regions ranked in order of highest mean connectivity. In both conditions region 4 (Skopje) has the highest connectivity. However, in the networks where landscape height modulated the virtual Plasmodium behaviour there is a shift in connectivity to regions to the left of region 1 (Thessaloniki). This is most evident with an increase in the connectivity of region 6 (Dyrrachium). The shift in region influence under the landscape height condition can be observed in the visualisations denoted in Fig. 15 where the radius of the regions vary in proportion to their mean degree value.

**Table 1:** Mean degree of region connectivity over 20 experiments ranked in descending order with no height representation (left) and with height representation (right).

**Region**	No heigh trepresentation		**Region**	With height representation	
	**Node**	Mean degree		**Node**	Mean degree
Scupi (Skopje) and/or Stobi	4	7.88	Scupi (Skopje) and/or Stobi	4	7.69
Thessaloniki	1	5.25	Dyrrachium	6	7.19
Remesiana–Naissus	13	5.19	Remesiana–Naissus	13	7
Philippopolis	15	4.94	Thessaloniki	1	6.31
Hadrianopolis	14	4.69	Serdica	10	6
Singidunum	16	4.69	Singidunum	16	5.69
Serdica	10	4.63	Hadrianopolis	14	5.44
Philippoi	2	4.13	Doclea	12	5
Doclea	12	4.06	Philippopolis	15	4.56
Dyrrachium	6	3.69	Nicopolis	3	4
Nicopolis	3	3.56	Philippoi	2	3.81
Traianopolis	5	2.81	Traianopolis	5	3.69
Heraclea	7	2.5	Heraclea	7	3.06
Marcianopolis	9	2.5	Constantinople/Byzantium	8	2.81
Constantinople/Byzantium	8	1.25	Marcianopolis	9	2.5
Sirmium	11	1.25	Sirmium	11	1.25
Tomis	17	1.25	Tomis	17	1.25

## Conclusions and Discussion

As has been repeatedly proved in different experiments (eg. experiments where slime mould navigates a maze) the simple amoeboid organism *P. Polycephalum* has an innate heuristic shortest path ability that can solve decision making problems (especially multi source problems). This becomes possible mainly due to the fact that the oscillatory cytoplasm of the Plasmodium is a spatially extended nonlinear excitable medium which closely matches the dynamics of other reaction – diffusion systems. As shown in the previous experiment^27^ the method can actually be applied on the archaeological or historical research as means to recreate networks and the decision making strategies behind them.

However, in order for the technique to evolve to a real archaeological tool, it must clearly be able to allow the researcher to incorporate in the experiments these geographical features which influenced or constrained choices important in the development of a network. The recent developments in 3D printing and solid terrain modelling promise a relatively accessible platform so as to test different case scenarios with the use of Physarum machines while at the same time Geoarchaeology^52^ can provide solid evidence so as to recreate terrains that will resemble very closely to past physical landscapes. It is clear that the heuristic ability of *P. Polycephalum* (and probably of similar unconventional computing methods) can evolve to become a very dynamic way to explore these terrains. Concerning the Roman road network in the Balkans the slime mould experiments on a 3D terrain showed that once more the *P. Polycephalum* managed to create a network which resembled to a great extent not only the results of the previous experiment on a flat terrain but also physical reality.

In our previous article we gave special emphasis on the interdisciplinary character of the experiment, noticing at the same time the limitations but also the theoretical considerations concerning the application of such methods in archaeology. The greatest possibly advantage of *P. Polycephalum* experiments as other unconventional computing methods lies on the fact that it provides a non human-biased method of modelling the world or living phenomena. 3D terrain brings the analogous modelling closer to reality since *P. polycephalum*, due to its positive geotropism, navigates around and through elevations recreating successfully the network. Overcoming this limitation (and the practical difficulties of applying a new computational method) it becomes clear that now the issue lies on the way that the technique can become a viable archaeological method. This requires the existence of a strong theoretical underpinning combined with well planned archaeological case studies which will instigate new experiments. Issues of specialized interest (for instance to explore the route that Via Egnatia followed in Thrace) or more theoretical and general questions (for instance the relationship between water bodies, river valleys and the development of roads) can be potentially be the next step for Physarum machines in archaeology.

## Electronic supplementary material

Supplementary information
