# Supplementary material for: Physarum machines imitating a Roman road network: the 3D approach
Source: Sci Rep. 2017 Aug 1;7:7010. doi: 10.1038/s41598-017-06961-y (PMC5539195; doi:10.1038/s41598-017-06961-y)
Supplement: Supplementary file 1 — Supplementary information [file 41598_2017_6961_MOESM1_ESM.pdf]

Supplementary Information:

Physarum machines imitating a Roman road network: the 3D approach

Vasilis Evangelidis[1], Jeff Jones[2], Nikolaos Dourvas[3], Michail-Antisthenis Tsompanas[2], Georgios Ch. Sirakoulis[3,*], and Andrew Adamatzky[2]

[1]Research and Innovation Centre “Athena”, Institute for Language and Speech Processing, Xanthi, GR67100, GREECE
[2]Unconventional Computing Centre, University of the West of England, Bristol BS16 1QY, United Kingdom
[3]Laboratory of Electronics, Department of Electrical and Computer Engineering, Democritus University of Thrace, Xanthi, GR67100, GREECE
[*]gsirak@ee.duth.gr

Particle Model Description

We used a multi-agent approach to generate the *Physarum*-like behaviour. This approach was chosen specifically because we wanted to reproduce the generation of complex behaviour using very simple component parts and interactions, and no special or critical component parts to generate the emergent behaviour. Although other modelling approaches, notably cellular automata, also share these properties, the direct mobile behaviour of the agent particles renders it more suitable to reproduce the flux within the Plasmodium. The multi-agent particle model of *Physarum* used to generate the transport networks[1] uses a population of coupled mobile particles with very simple behaviours, residing within a 2D diffusive lattice. The lattice stores particle positions and the concentration of a local diffusive factor referred to generically as chemoattractant. Particles deposit this chemoattractant factor when they move and also sense the local concentration of the chemoattractant during the sensory stage of the particle algorithm. Collective particle positions represent the global pattern of the material. The model runs within a multi-agent framework running on a Windows PC system. Performance is thus influenced by the speed of the PC running the framework. The particles act independently and iteration of the particle population is performed randomly to avoid any artifacts from sequential ordering.

Generation of Virtual Plasmodium Networks

The behaviour of the particles occurs in two distinct stages, the sensory stage and the motor stage. In the sensory stage, the particles sample their local environment using three forward biased sensors whose angle from the forwards position (the sensor angle parameter, *SA*), and distance (sensor offset, *SO*) may be parametrically adjusted (Supplementary Fig. 1A). The offset sensors generate local indirect coupling of sensory inputs and movement to generate the cohesion of the material. The *SO* distance is measured in pixels and a minimum distance of 3 pixels is required for strong local coupling to occur. For the experiments in this article we used an *SO* value of 5. During the sensory stage each particle changes its orientation to rotate (via the parameter rotation angle, *RA*) towards the strongest local source of chemoattractant (Supplementary Fig. 1B). Variations in both *SA* and *RA* parameters have been shown to generate a wide range of reaction-diffusion patterns[2] and for these experiments we used *SA*=60 and *RA*=60 which results in adaptation and minimisation of the virtual transport networks. After the sensory stage, each particle executes the motor stage and attempts to move forwards in its current orientation (an angle from 0–360 degrees) by a single pixel forwards. Each lattice site may only store a single particle and particles deposit chemoattractant into the lattice (5 units per step) only in the event of a successful forwards movement. If the next chosen site is already occupied by another particle move is abandoned and the particle selects a new randomly chosen direction.

Environment and Problem Data Representation

Diffusion of the particle trails in the 2D lattice was implemented at each scheduler step, and at every site in the lattice, via a simple mean filter of kernel size 3×3. Damping of the diffusion field, which limits the distance of chemoattractant gradient diffusion, was achieved by multiplying the mean kernel value by 0.9 per scheduler step. The spatially implemented computation requires that the data configuration be contained within the 2D lattice containing the particle population. Data configurations of the Balkans region boundaries are loaded as greyscale image files and this data is interpreted by the scheduler and projected into the diffusive lattice. Specific region site positions are loaded from a text file and represented as a virtual chemoattractant

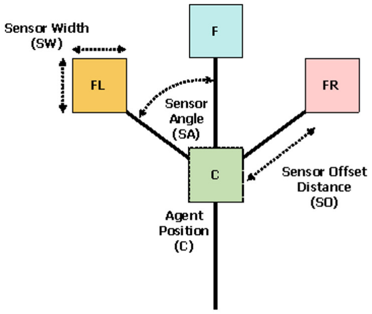


```
[Sensory stage]

- Sample chemoattractant map values
- if (F > FL) && (F > FR)
      - Continue facing same direction
- Else if (F < FL) && (F < FR)
      Rotate by RA towards larger of FL and FR
- Else if (FL < FR)
      Rotate right by RA
- Else if (FR < FL)
      Rotate left by RA
- Else
      Continue facing same direction
```

(a) (b)

Supplementary Figure 1. Architecture of a single particle of the virtual material and its sensory algorithm. A: Morphology showing agent position 'C' and offset sensor positions (FL, F, FR), B: Algorithm for particle sensory stage.

projected into the lattice (2.55 units per scheduler step) to attract local particles. For the condition where landscape terrain height is represented, the heightfield map is stored as an greyscale image where pixel intensity corresponds to landscape height (zero is minimum height, 255 is maximum height). The landscape height modulates agent sensor values in the following way: The average landscape height in a 9×9 window, centred at each sensor location, is sampled and normalised by dividing by 255 to give a height value, h between zero and 1. Each separate lattice sensor value (sampling from the diffusive lattice, representing the virtual network) is multiplied by $1 - h$ to modulate its value. The result is that sensor values are reduced more strongly at large landscape heights, as opposed to small landscape heights.

Network Adaptation and Minimisation Mechanism

Adaptation and minimisation of the virtual transport networks is implemented via tests executed at regular intervals as follows. If there are 1 to 10 particles in a 9×9 neighbourhood of a particle, and the particle has moved forwards successfully, the particle attempts to divide into two if there is a space available at a randomly selected empty location in the immediate 3×3 neighbourhood surrounding the particle. If there are 0 to 24 particles in a 5×5 neighbourhood of a particle the particle survives, otherwise it is deleted. Deletion of a particle leaves a vacant space at this location which is filled by nearby particles, causing the collective to shrink slightly. As the process continues the network shrinks and adapts its morphology to the stimuli provided by the configuration of Balkans region stimuli (if no external stimuli were present the material would eventually adapt to a minimal circular shape and shrink down to a small cluster of points in size). The frequency at which the growth and shrinkage of the population is executed determines a turnover rate for the particles. The frequency of testing for particle division and particle removal was every 3 scheduler steps.

Halting Mechanism and Network Analysis

Experiments were halted after 20,000 scheduler steps. This amount of running time enables the networks to reach a stable state. During the experiments, snapshots of the network configuration were saved at regular intervals. The final image at 20,000 steps was used to analyse the network connectivity. We analysed network connectivity using the method described in[3]. This analysis produced adjacency matrices describing the final connectivity of the networks.

References

1. Jones, J. The emergence and dynamical evolution of complex transport networks from simple low-level behaviours. *Int. Journal of Unconventional Comput.* **6**, 125–144 (2010).
2. Jones, J. Characteristics of pattern formation and evolution in approximations of *Physarum* transport networks. *Artificial Life* **16**, 127–153 (2010).
3. Jones, J. Towards programmable smart materials: Dynamical reconfiguration of emergent transport networks. *Int. Journal of Unconventional Computing* **7**, 423–447 (2011).
